# The genus *Micatagla* Argaman, 1994 in Egypt, with three new species and a new record (Hymenoptera, Bradynobaenidae, Apterogyninae)

**DOI:** 10.3897/zookeys.397.6768

**Published:** 2014-04-03

**Authors:** Neveen S. Gadallah, Ahmed M. Soliman

**Affiliations:** 1 Department of Entomology, Faculty of Science, Cairo University, Giza, Egypt; 2 Department of Zoology, Faculty of Science (Boys), Al-Azhar University, Nasr City, Cairo, Egypt

**Keywords:** Apterogyninae, *Micatagla*, new species, new record, faunistic list

## Abstract

The genus *Micatagla* Argaman (Bradynobaenidae: Apterogyninae) is reviewed from Egypt, based on specimens collected from Wadi Allaqi (Aswan, Southern Egypt) and Kom Osheim (Fayoum) and those deposited in Egyptian insect collections as well as recorded data from the literature. A single species, *Micatagla klugi* (André), was previously recorded from Egypt. *Micatagla allaqiensis* **sp. n.**, *Micatagla ezzati* **sp. n.** and *Micatagla pseudorainerii* **sp. n.** are described here. *Micatagla antropovi* Pagliano is also newly recorded from the Egyptian fauna. An illustrated key and a faunistic list comprising all *Micatagla* species recorded from Egypt are given.

## Introduction

The genus *Micatagla* Argaman, 1994 is a relatively large genus in the subfamily Apterogyninae, with 47 recorded species (Pagliano 2002). It was first erected by Argaman (1994) with only a single species (female), *Micatagla schulzei* (André, 1909) from Namibia. Members of the genus are widely distributed in Africa; only two species were recorded from Asia (Pagliano 2002). Their biology is still unknown.

Members of the genus *Micatagla* are characterized by their small to medium size, 4–18 mm long; third metasomal tergite (T3) of female without basal tegumental yellow spot; eyes (female) small, distant from occipital carina at least by their own diameter; hind trochanter (male) with ventral lamella; forewing (male) with closed brachial cell (except open in *Micatagla noorti*). Both sexes are normally quite colourful as they consist of red mixed with ferruginous to black, only few species have individuals all black.

In Egypt, the genus *Micatagla* is represented by a single species, *Micatagla klugi* (André, 1899) (Pagliano 2002). In the present study, three new species: *Micatagla allaqiensis*, *Micatagla ezzati* and *Micatagla pseudorainerii* are described and illustrated, and a new record, *Micatagla antropovi* Pagliano, 2002, is also added to the Egyptian fauna thus increasing the total number to five species. An illustrated key for identifying all the Egyptian species is also given.

## Material and methods

The present study is based on specimens collected from Wadi Allaqi (Aswan, Southern Egypt) and Kom Osheim (Fayoum) and those deposited in the Egyptian insect collections as well as previous records from Egypt. Sampling was done by means of pitfall traps. Morphological terms are based on Pagliano (2002). Body-sculpture terminology is based on Harris (1979). Photos were taken by Canon camera (G12), attached to Optech trinocular zoom stereomicroscope (LFZT). The distribution of *Micatagla* species in the different Egyptian localities is plotted (Fig. 31) using DIVA-GIS (Ver.7.1.7). The type specimens of the new species are deposited in the Efflatoun Bey collection, Entomology Department, Faculty of Science, Cairo University, Giza (Egypt) (CUE).

**Collection sites.** Aswan: 24°05'26"N, 32°54'00"E; Bir Um Reiga: 29°32'28"N, 32°21'45"E; Kafr Hakim: 30°04'54"N, 31°07'00"E; Kom Osheim: 29°33'46"N, 30°54'36"E; Massara: 30°04'15"N, 31°14'43"E; Mokattam: 30°01'00"N, 31°17'16"E; Wadi Allaqi: 22°50'20"N, 33°11'54"E; Wadi Assiouti: 27°12'30"N, 31°18'50"E; Wadi Digla: 29°57'30"N, 31°20'06"E; Wadi el-Tih: 29°09'00"N, 33°32'00"E; Wadi Garawi: 29°48'02"N, 31°27'39"E. Wadi Hoff: 29°53'22"N, 31°20'25"E.

**Collection repositories** (abbreviations based on Evenhuis 2012). BMNH = The Natural History Museum, London, United Kingdom; CUE = Efflatoun Bey collection, Entomology Department, Faculty of Science, Cairo University, Giza, Egypt; ESEC = Egyptian Entomological Society collection, Cairo, Egypt; MNHN = Muséum National d’Histoire Naturelle, Paris, France; MSNG = Museo Civico di Storia Naturale ’G. Doria’, Genoa, Italy; PPDD = Ministry of Agriculture collection, Giza, Egypt.

**Abbreviations.** F1, F2, F3, etc. = first, second, third, etc., antennal flagellomeres; S1, S2, S3, etc. = first, second, third, etc., metasomal sternites; T1, T2, T3, etc. = first, second, third, etc., metasomal tergites.

## Results and discussion

### Key to the females of the Egyptian species of the genus *Micatagla* Argaman

**Table:** 

1	Both first and second metasomal segments red (Figs 7, 13, 19)	2
–	First metasomal segment red, the second black (Figs 1, 25)	4
2	Mesosomal dorsum, T2 & T3 closely regularly longitudinally striate (Figs 9, 11)	*Micatagla klugi* (André)
–	Mesosomal dorsum punctate, T2 foveolate, T3 closely longitudinally striate	3
3	Body length 5.5 mm; malar space slightly longer than longitudinal eye diameter; mid and hind coxae entirely red; T1 superficially punctate, T2 with ellipsoid foveae separated by longitudinal ridges (Fig. 17), T3 finely and closely striate longitudinally; T6 reddish, with darker interrupted carinae (Fig. 18)	*Micatagla pseudorainerii* sp. n.
–	Body length 8 mm; malar space slightly shorter than longitudinal eye diameter; mid and hind coxae red, with yellow apices; T1 deeply foveate, T2 and T3 with ellipsoid foveae separated by longitudinal ridges (Fig. 23); T6 dark brown with black interrupted carinae (Fig. 24)	*Micatagla allaqiensis* sp. n.
4	Body length 8 mm; red of the body dark; mandible bidentate sub-apically; T2 and T3 black with interrupted ridges forming deep widely spaced ellipsoid punctures (Fig. 5); T6 black, bordered with well developed sharp teeth (Fig. 6); S2 and S3 deeply punctate-reticulate	*Micatagla antropovi* Pagliano
–	Body length 6 mm; red of the body light; mandible edentate; T2 and T3 blackish red (Fig. 29); T2 with ellipsoid punctures and longitudinal shiny ridges in between (Fig. 29); T3 with fine longitudinal regular ridges (Fig. 29); T6 brownish red, bordered with less developed blunt teeth (Fig. 30); S2 and S3 with widely spaced punctures	*Micatagla ezzati* sp. n.

### Egyptian species of the genus *Micatagla* Argaman

#### Micatagla antropovi

Pagliano, 2002

http://species-id.net/wiki/Micatagla_antropovi

Figs 1–6

Micatagla antropovi Pagliano, 2002: 222, holotype ♀: Abu Arish (Saudi Arabia) (BMNH).

##### Material examined.

Egypt, 1 ♀, Kom Osheim (Fayoum), 30.v.2013 (leg. Ahmad M. Soliman) [CUE].

**Figures 1–6.**
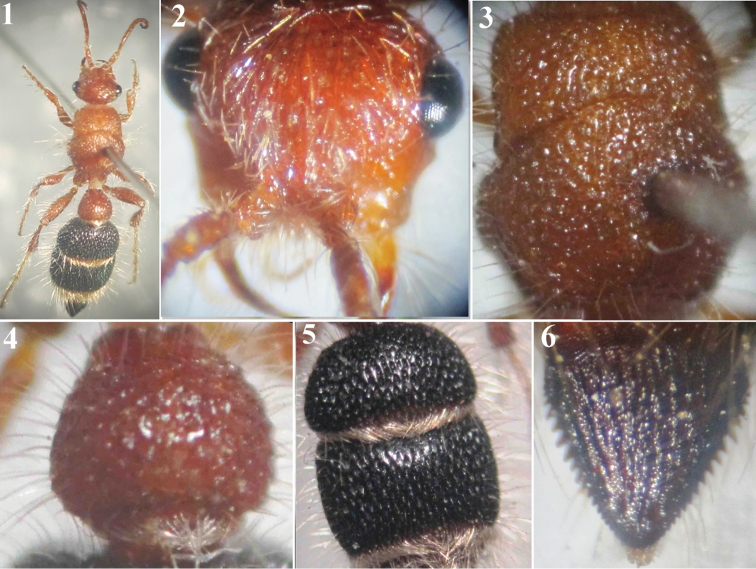
*Micatagla antropovi* Pagliano, female: **1** Habitus, dorsal view **2** Head, frontal view **3** Mesosoma, dorsal view **4** T1 **5** T2 & T3 **6** T6.

##### Distribution.

Saudi Arabia, Yemen (Pagliano 2002); Egypt (new record).

#### Micatagla klugi

(André, 1899)

http://species-id.net/wiki/Micatagla_klugi

Figs 7–12

Apterogyna klugi André, 1899: 69, holotype ♀: Aswan (Egypt), (probably in MNHN).
Apterogyna klugi : André 1910: 16, ♀; Bischoff 1920: 48, ♀; Invrea 1951: 167, ♀; Invrea 1963: 14, ♀; Invrea 1965: 55, ♀ (? ♀ of *mocsaryi*).
Apterogyna gridellii Invrea, 1959: 117, holotype ♀: Egypt; synonymized with *Micatagla klugi* by Pagliano 2002: 269.
Utapitoca klugi : Argaman 1994: 48, ♀ & ♂ (♂ not described)

##### Material examined.

Egypt, 8 ♀, Wadi Assiouti (Assiout), 1–2.iv.1917 (leg. André) [PPDD]; 1♀, Cairo, with no date (leg. Adair) [PPDD]; 1♀, Mokattam (Cairo), April (leg. Innes Bey) [ESEC]; 1♀, without locality or date (leg. Ferrant) [ESEC].

**Figures 7–12.**
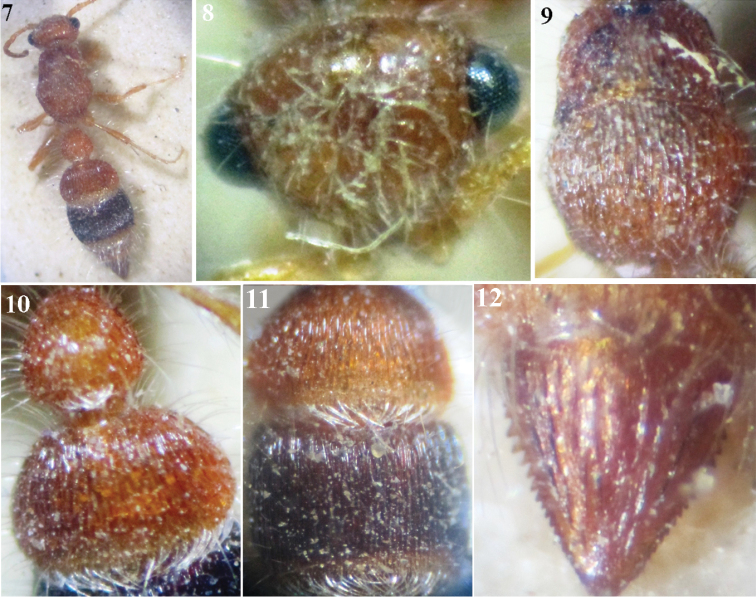
*Micatagla klugi* (André), female: **7** Habitus, dorsal view **8** Head, frontal view **9** Mesosoma, dorsal view **10** T1 & T2 **11** T2 & T3 **12** T6.

##### Previous records from Egypt.

Wadi Hoff (Helwan), Bir Um Reiga (Red Sea), Massara (Cairo), Wadi Digla (Cairo), Wadi Garawi (Helwan), Wadi el-Tih (Sinai) (Pagliano 2002).

##### Distribution.

Egypt; Saudi Arabia (Pagliano 2002).

#### Micatagla pseudorainerii

Gadallah & Soliman sp. n.

http://zoobank.org/EACEA2EE-E24B-49DB-8F48-64465128B1D5

http://species-id.net/wiki/Micatagla_pseudorainerii

Figs 13–18

##### Material examined.

Holotype ♀: Egypt, Wadi Assiouti, Assiout [27°12'30"N, 31°18'50"E], 13.iv.1934 (leg. ?) [CUE].

##### Description.

**Female (Holotype).** Body length 5.5 mm.

**Colour.** Red, except T3 dark ferruginous to black with red posterior margin, T1 slightly darker than the rest of tergites, S1 and S3 dark ferruginous, S2 slightly dark red, T6 with dark red interrupted longitudinal ridges.

**Pubescence.** Body and legs densely clothed with fine whitish hairs; head with some hairs, along face, that are erect and longer around eyes; mesosomal tergites with few hairs along their dorsal surfaces, posterior margin of all tergites with fringes of dense, inwardly directed silvery hairs that are crossing centrally.

**Head** (Fig. 14). In dorsal view, slightly wider than pronotum, with some scattered superficial punctures, far apart by about 2–4 times their diameter; vertex slightly semi-circular to flattened; eye small, prominent, located above midline between clypeus and vertex when seen from frontal view; distance between antennal tubercles slightly longer than tubercle length; malar space relatively long, slightly longer than longitudinal eye diameter. Scape of antenna 2.5× as long as F1, gently convex from above, flagellomeres polished, F1 about as long as F2.

**Figures 13–18.**
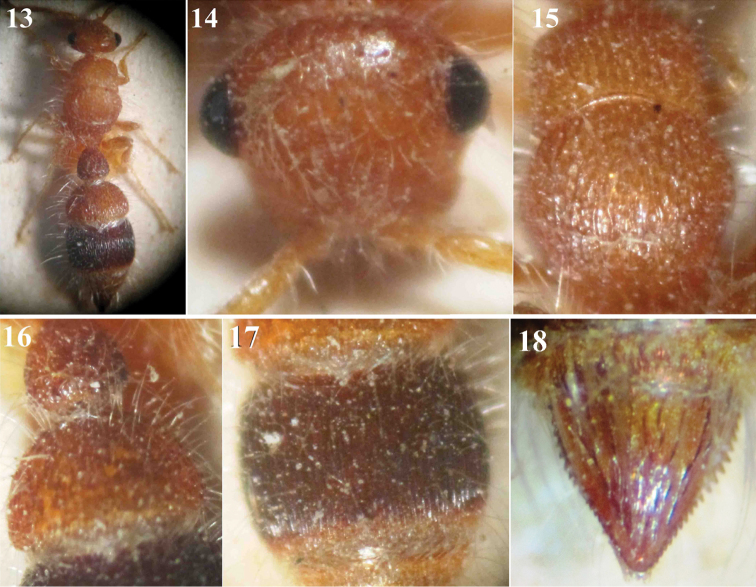
*Micatagla pseudorainerii* Gadallah & Soliman, sp. n., holotype female: **13** Habitus, dorsal view **14** Head, frontal view **15** Mesosoma, dorsal view **16** T1 & T2 **17** T3 **18** T6.

**Mesosoma** (Fig. 15). Pronotum quadrate, about as long as maximum width, with gently declivous anterior face, rounded humeral angle and slightly concave to straight posterior margin; densely foveate-reticulate dorsally; remainder of mesosomal dorsum superficially punctuate, punctures are somewhat spaced by a distance equal to their own diameter. Propodeal posterior face gently declivous, smooth and impunctate. Propleuron faintly punctured, with some incomplete longitudinal separated ridges posteriorly; mesopleuron foveate-reticulate; metapleuron smooth. Hind tibial spurs about equal in length.

**Metasoma.** T1 widened posteriorly (pear-shaped), as long as its maximal width (Fig. 16), with some dispersed punctures hidden behind hairs; T2 with ellipsoid foveae separated by longitudinal ridges (Fig. 17); T3 finely and closely striated longitudinally; T6 (pygidium) subtriangular, with longitudinal interrupted widely spaced carinae, bordered laterally with small sharp teeth that progressively reduce in size and become blunt distally, apical ones rounded (Figs 24, 25). Metasomal sternites smooth, with some scattered, erect fine hairs in the middle longitudinally.

**Figures 19–24.**
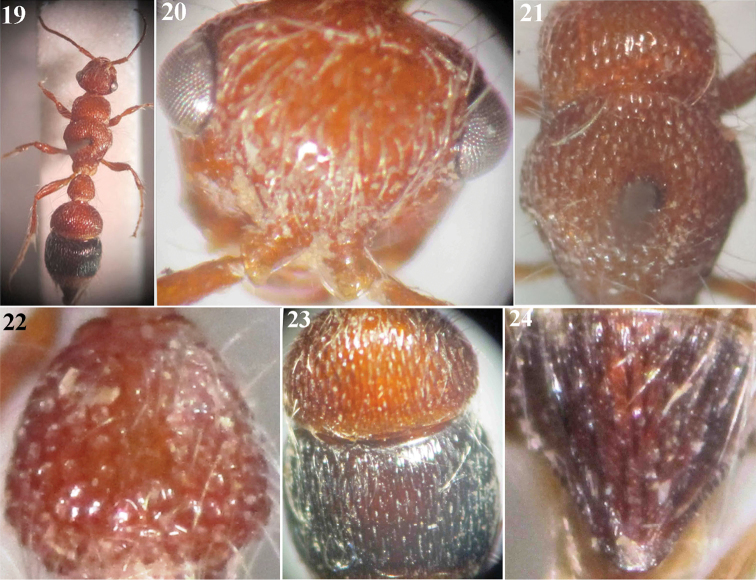
*Micatagla allaqiensis* Gadallah & Soliman, sp. n., holotype female: **19** Habitus, dorsal view **20** Head, frontal view **21** Mesosoma, dorsal view **22** T1 **23** T2 & T3 **24** T6.

**Figures 25–30.**
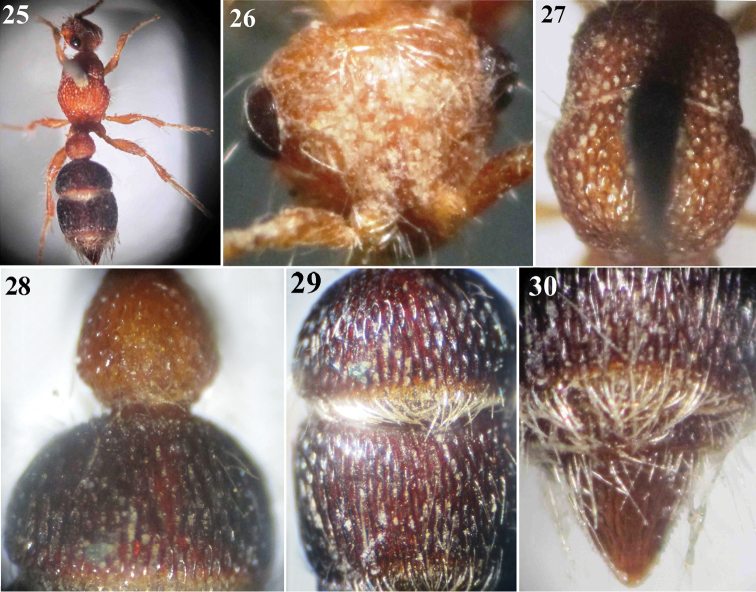
*Micatagla ezzati* Gadallah & Soliman, sp. n., holotype female: **25** Habitus, dorsal view **26** Head, frontal view **27** Mesosoma, dorsal view **28** T1 & T2 **29** T2 & T3 **30** T6.

**Figure 31.**
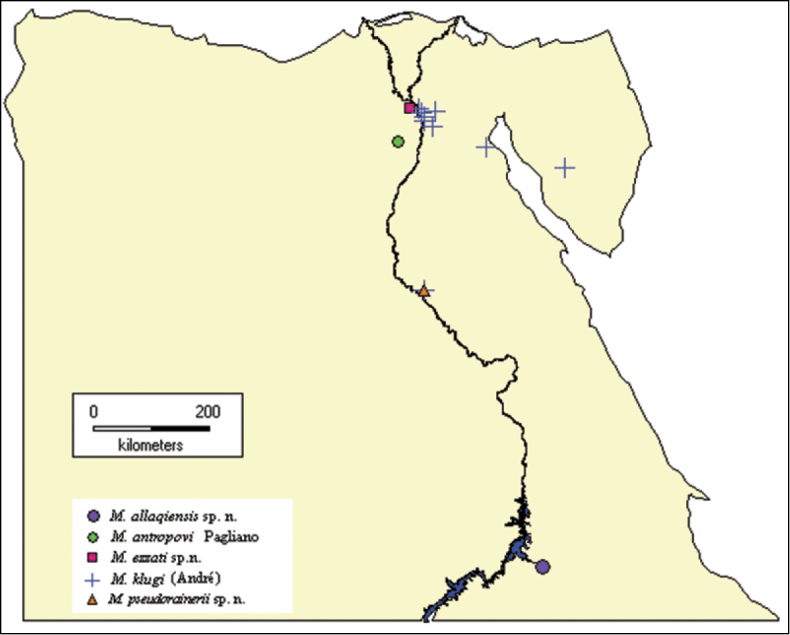
Distributional map of *Micatagla* species in Egypt.

##### Remarks.

*Micatagla pseudorainerii* is nearest to *Micatagla rainerii* Pagliano, 2002 which is found in Namibia, and the two species are distinguished by the following characters:

**Table:** 

1	Body length 5.5 mm; metasomal tergites with distinct dense fasciae of silvery bristle-like hairs at posterior margin, inwardly directed and crossed centrally; S2 without sub-lateral blackish spots; mandibles entirely red	*Micatagla pseudorainerii* sp. n.
–	Body length 3.5 mm; metasomal tergites with few silvery hairs at posterior margins; S2 with sub-lateral blackish spots; mandibles brownish at distal half	*Micatagla rainerii* Pagliano

##### Etymology.

The name *pseudorainerii* refers to the similarity of this species to *Micatagla rainerii*.

#### Micatagla allaqiensis

Gadallah & Soliman sp. n.

http://zoobank.org/A20109F5-2355-42D5-A6B7-7F11F2B00598

http://species-id.net/wiki/Micatagla_allaqiensis

Figs 19–24

##### Material examined.

Holotype ♀: Southern Egypt, Wadi Allaqi, Aswan [22°50'20"N, 33°11'54"E], 20.xii.2012 (leg. Ahmed M. Soliman) [CUE].

##### Description.

**Female (Holotype).** Body length 8 mm.

**Colour.** Medium yellowish brown, except antennal flagellum brown, mandible reddish brown distally; fore tibial spur light red, mid and hind tibial spurs waxy white; mid and hind coxae with yellow apices; T2 with apical yellow band widened medially, T3 black with apical yellowish band, S3 black on basal two-thirds, pygidial area dark brown with black carinae.

**Pubescence.** Body as well as legs rather densely clothed with erect to suberect white hairs (recumbent on face, denser on clypeus and metasomal segments 4–5 than elsewhere). T1–3 with apical fringe of compact inward directed white hairs, that is weak on T1; S2 and S5 with apical fringe of obviously scattered white hairs.

**Head.** (Fig. 20): In dorsal view a little wider than pronotum and abruptly converging behind eyes; vertex gently sloping posteriorly; occiput feebly convex; vertex, face and gena shallowly sparsely punctate, punctures on gena more dispersed; ocular orbit deeply punctate; eye small, subspherical and prominent, located above midline between upper margin of clypeus and vertex; malar space and antennal scrobe polished; distance between antennal tubercles noticeably shorter than tubercle length; clypeus strongly bent downward, polished apically, punctate basally; gena with obsolescent obtuse tubercle behind eye immediately below lower ocular margin; malar space markedly long (slightly shorter than longitudinal eye diameter); mandible slender, sickle-shaped, edentate; palpi with long and slender segments. Scape of antenna 2.35× as long as F1, gently and evenly convex from above; flagellomeres polished, F1 scarcely longer than F2, F2 as long as F3, F2–7 flattened beneath.

**Mesosoma** (Fig. 21). Pronotum 0.4× as long as its maximal width, with anterior face abruptly declivous, humeral angle gently rounded and posterior margin broadly concave; dorsally deeply foveate-reticulate; horizontally carinate laterally; remainder of mesosomal dorsum foveate-striate. Propodeal posterior face gently declivous and impunctate. Mesopleuron deeply foveate-reticulate; metapleuron faintly horizontally carinate. Mesosternum strongly bidentate in front of hind coxae. Inner hind tibial spur long (0.85× as long as basal tarsomere).

**Metasoma.** T1 (Fig. 22) widened posteriorly (pear-shaped), as long as its maximal width, densely foveate, abruptly declivous along posterior rim; T2 semicircular, 0.6× as long as wide, strongly petiolate anteriorly; T3 narrowed anteriorly forming a constriction with T2; T2 and T3 with ellipsoid foveae separated by longitudinal ridges (Fig. 23), foveae on T3 slimmer than those on T2, ridges on apical fourth of T3 fine and closer to each other than elsewhere; T6 (pygidium) subtriangular, with longitudinal interrupted widely spaced carinae, bordered laterally with small sharp teeth that progressively reduce in size and become blunt distally, apical ones rounded (Fig. 24). S1 smooth, transversely carinate basally; S2 deeply punctate-reticulate (impunctate on anterior declivity and posteromedially); S3 densely punctate laterally, impunctate medially; S4 and S5 with few scattered punctures; S2–5 with a row of punctures along their apical margins.

##### Etymology.

The specific name originates from Wadi Allaqi (Aswan, southern Egypt), the type locality.

#### Micatagla ezzati

Gadallah & Soliman sp. n.

http://zoobank.org/F10F2B49-17A0-4EBA-B90B-548C19DB6DA3

http://species-id.net/wiki/Micatagla_ezzati

Figs 25–30

##### Material examined.

Holotype ♀: Egypt, Kafr Hakim, Giza [30°04'54"N, 31°07'00"E], 8.v.1932 (leg.?) [CUE].

##### Description.

**Female (Holotype).** Body length 6 mm.

**Colour.** Red, except mandible reddish brown distally, maxillary and labial palpi pale; mid and hind tibial spurs waxy white; 2^nd^ & 3^rd^ metasomal segments blackish red (posterior margin of T2 & T3 whitish, slightly widened medially), T4 & T5 pale red, T6 reddish brown.

**Pubescence.** Body including legs clothed with fine erect to recumbent whitish hairs, relatively longer on mesopleuron and distinctly denser on face and vertex of head, T4 and T5 than elsewhere. Posterior margin of T1 with fringe of irregular and inwardly directed whitish hairs; T2 and T3 with apical fascia of silvery inwardly directed hairs that are much denser than that of T1.

**Head** (Fig. 26). In dorsal view slightly wider than pronotum and strongly convergent behind eyes; face and vertex clothed with recumbent whitish hairs masking the sculpturing beneath; vertex gently sloping posteriorly; eye small, subspherical and strongly prominent, located above midline between clypeal free margin and vertex; antennal scrobe polished; distance between antennal tubercles equal to tubercle length; clypeus bent downward, polished apically, with straight free margin, punctate basally; gena very sparsely punctate, with noticeable obtuse tubercle behind eye immediately below lower ocular margin; malar space relatively long, as long as longitudinal eye diameter; mandible slender, edentate; palpi with long and slender segments. Scape of antenna 2.5× as long as F1, gently and evenly convex from above; flagellomeres polished, F1 scarcely longer than F2, F2 as long as F3, F2–7 flattened beneath.

**Mesosoma** (Fig. 27). Pronotum about 0.4× as long as its maximum width, with gently declivous anterior face, rounded humeral angle and broadly concave posterior margin; foveate-reticulate dorsally, faintly striated laterally; remainder of mesosomal dorsum foveate-striate. Propodeal posterior face gently declivous and impunctate. Mesopleuron shallowly foveate-reticulate; metapleuron smooth. Inner hind tibial spur long (0.75× as long as basal tarsomere).

**Metasoma.** T1 (Fig. 28) gradually widened posteriorly (pear-shaped), as long as its maximal width, shallowly and coarsely areolate, abruptly declivous posteriorly; T2 semicircular, 0.6× as long as wide, petiolate anteriorly, with ellipsoid punctures separated by longitudinal shiny ridges (Fig. 29); T3 with fine longitudinal regular ridges (Fig. 29); T6 (pygidium) subtriangular, with dark interrupted, widely separated, longitudinal carinae, bordered with small sharp teeth spaced in the middle, becoming smaller, blunter and closer distally (Fig. 30). S1 bare, moderately punctured; S2 and S3 shallowly punctate-subreticulate, punctures dispersed on the disc of both sternites, S3 impunctate subapically; S2–5 punctate along apical margins.

##### Etymology.

This species is named in honour of the late Prof. Yahia Ezzat (the professor of the first author).

## Supplementary Material

XML Treatment for Micatagla antropovi

XML Treatment for Micatagla klugi

XML Treatment for Micatagla pseudorainerii

XML Treatment for Micatagla allaqiensis

XML Treatment for Micatagla ezzati
